# A Vertical Single Transistor Neuron with Core–Shell Dual-Gate for Excitatory–Inhibitory Function and Tunable Firing Threshold Voltage

**DOI:** 10.3390/mi13101740

**Published:** 2022-10-14

**Authors:** Taegoon Lee, Seung-Bae Jeon, Daewon Kim

**Affiliations:** 1 Department of Electronic Engineering, Kyung Hee University, 1732 Deogyeong-daero, Giheung-gu, Yongin 17104, Korea; 2 Department of Electronic Engineering, Hanbat National University, 125 Dongseo-daero, Yuseong-gu, Daejeon 34158, Korea

**Keywords:** low-power electronics, neuromorphic computing, artificial neurons, leaky integrate-and-fire (LIF) neuron, homeostasis, neuron inhibition, spiking neural network (SNN)

## Abstract

A novel inhibitable and firing threshold voltage tunable vertical nanowire (NW) single transistor neuron device with core–shell dual-gate (CSDG) was realized and verified by TCAD simulation. The CSDG NW neuron is enclosed by an independently accessed shell gate and core gate to serve an excitatory–inhibitory transition and a firing threshold voltage adjustment, respectively. By utilizing the shell gate, the firing of specific neuron can be inhibited for winner-takes-all learning. It was confirmed that the independently accessed core gate can be used for adjustment of the firing threshold voltage to compensate random conductance variation before the learning and to fix inference error caused by unwanted synapse conductance change after the learning. This threshold voltage tuning can also be utilized for homeostatic function during the learning process. Furthermore, a myelination function which controls the transmission rate was obtained based on the inherent asymmetry between the source and drain in vertical NW structure. Finally, using the CSDG NW neuron device, a letter recognition test was conducted by SPICE simulation for a system-level validation. This multi-functional neuron device can contribute to construct a high-density monolithic SNN hardware combining with the previously developed vertical synapse MOSFET devices.

## 1. Introduction

Neuromorphic computing architecture has been intensively studied to overcome the bottlenecks of the conventional von Neumann computing architecture, such as limitations of maximum processing speeds and high levels of energy consumption [1,2,3,4]. Especially, a spiking neural network (SNN) which mimics the biological spike-based learning has attracted great deal of attentions due to its low power consumption [5,6]. The SNN hardware demands a neuron device that encodes signals into a spike-shaped signal. Among various neuron models, a leaky integrate-and-fire (LIF) neuron model that integrates the signals from pre-synapses and fires a spike signal to the next neurons when the integrated signals exceed a threshold has been intensively studied [7,8,9]. Recently, a gate-less LIF neuron device was developed by use of a biristor which shows two resistance states based on the single transistor latch (STL) phenomenon [7]. In this neuron model, charges from applied input current are accumulated at the drain node under the initial high resistance state (HRS) because of the high potential barrier across the channel. When accumulated charges exceed a certain level, high potential barrier was lowered by the STL phenomenon and resistance state was shifted from the HRS to a low resistance state (LRS). At this moment, the accumulated charges are discharged while generating voltage spikes. In addition to simple signal encoding and transmission, few attempts have been dedicated to imitating some beneficial functions of a biological neuron, such as inhibition, firing threshold voltage tuning, and myelination in artificial LIF neuron model. The inhibition function which selectively disables the firing of specific neurons was realized using a MOSFET based single transistor neuron device to improve the power efficiency of neuromorphic system and promote an effective learning via a winner-takes-all (WTA) mechanism [10,11,12,13,14]. This inhibition function is commonly achieved by tuning of firing threshold voltage to a very high level in MOSFET based devices. Apart from complete inhibition, fine tuning of firing threshold voltage is also required in SNN for many circumstances. For example, synaptic devices connected to the neuron devices can suffer from the endurance problems and variations arisen from short channel effects, band-to-band tunneling (BTBT), and fabrication process [15]. In that case, the SNN can make a wrong decision during the inference. By the firing threshold voltage tuning of a specific neuron, this kind of error can be compensated [16]. Additionally, homeostasis function [17,18,19] can be served by threshold voltage tuning while the learning is being operated. Lastly, a myelination function which controls transmission rate of information by changing the membrane capacitance using myelin sheath was realized in artificial neuron device to control computing speed and power consumption of neuromorphic system [20].

For chip level development of SNN, apart from the electrical characteristics and aforementioned key functions of a single neuron device, co-integration of the neuron and synapse devices should be considered. The SNN hardware comprises of neuron and synapse devices which use different materials and not compatible processes, can suffer from the extra energy consumption at the interface and reduced packing density owing to the interconnections. Therefore, it is advantageous that neuron and synapse devices have same structures and materials to enable monolithic integration. In earlier research, planar-typed neuron and synapse devices with the same silicon–oxide–nitride–oxide–silicon (SONOS) structure were demonstrated for the co-integration [21]. It has been verified that a footprint area of the neuron device can be reduced to 6 F^2^. This footprint size can be scaled further if a vertical-typed device is employed. Therefore, a neuron device with a footprint which is smaller than 4F^2^ have been developed by using a gate-all-around (GAA) vertical-typed single transistor [20]. In this sense, a vertical synapse device which can be co-integrated with the vertical neuron device was demonstrated employing core–shell dual-gate nanowire structure [15]. Although a small footprint area has been achieved and the potential for dense co-integration of neuron and synapse devices has been checked in the previous studies [20], the realization of all the aforementioned key functions, such as inhibition, threshold voltage tuning, and myelination in one vertical-typed neuron device has not been sufficiently investigated in the research field of neuromorphic hardware. If one vertical-typed neuron device could not serve all the aforementioned key neuronal functions, additional add-on devices are required to serve each key function, and this diminishes downscaling effect of a vertical-typed structure. Therefore, it is still needed to improve the vertical neuron device to serve various key neuronal functions while maintaining the small footprint and compatible structure for the co-integration with the synapse device.

This work suggests a vertical core–shell dual-gate (CSDG) nanowire (NW) structure based single transistor neuron with excitatory–inhibitory switching, threshold voltage tuning, and myelination functions. Since the overall structure and materials are same with the previously developed vertical synapse device [15], it is expected that the proposed neuron device can be a powerful candidate for a foundation of high-density monolithic SNN hardware. Device design, optimization and verification of various neuronal functions were conducted using TCAD simulation. The neuron device is enclosed by core–shell dual-gates. The shell gate with a nitride charge trapping layer was used for excitatory–inhibitory (E/I) function by modulating a potential barrier. In earlier research, it has been verified that the firing threshold voltage can be tuned by controlling the trapped charge density in nitride layer under the single shell (outer) gate [21]. However, this single gate may receive the signals from prior neuron or a signal for excitatory–inhibitory transition simultaneously in practical applications and there could be some disturbs. To address this problem, an independently accessed extra core (inner) gate was implemented in the proposed neuron device to receive firing threshold voltage adjustment signal. Independent operations of E/I function and firing threshold voltage adjustment in vertical-typed dual-gate neuron device were confirmed. On the other hand, it was verified that the core gate can be applied for various applications requiring tunable firing threshold voltage, such as an inference error fixation and compensation of random process variation of synapse. Using the inherent asymmetry between the drain and source in vertical NW structure, the myelination function was also realized. Finally, a simple system-level validation was conducted by letter recognition test via SPICE simulation reflecting the device characteristics of the proposed neuron device.

## 2. Device Structure and Simulation Methodology

**Figure 1:**
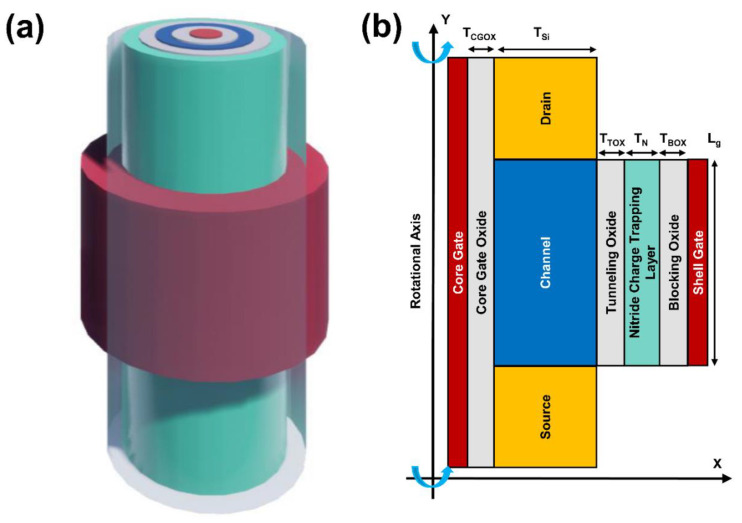
(**a**) The schematic image and (**b**) the cross-sectional view of a core–shell dual-gate nanowire neuron.

Figure 1a,b show the schematic image and cross-sectional view of a vertical CSDG NW neuron device. The device consists of two independently accessed double gates (inner core gate and outer shell gate) in gate all around manner and can be fabricated by the bottom-up processes [22]. The proposed device has a vertical silicon (Si) NW with a n^+^ drain (D) at the top and a n^+^ source (S) at the bottom of the p type body. The shell gate length (*L_g_*) and the Si channel thickness (*T_Si_*) were fixed to 500 nm and 200 nm, respectively. In vertical Si NW structure, an asymmetric doping concentration is unavoidable due to the different ion implantation energies for the top drain and bottom source regions [23]. Therefore, the doping concentrations of channel, source, and drain were modeled to 1 × 10^17^ cm^−3^, 5 × 10^19^ cm^−3^, and 1 × 10^20^ cm^−3^, respectively. Furthermore, in vertical Si NW structure, the size of a common source region is inherently larger than a tip drain region [20]. Based on the inherent asymmetry of source and drain, the parasitic capacitance of drain (0.41 fF) was modeled to be smaller than source (0.51 fF). The neuron device includes outer shell gate and inner core gate. The outer shell gate with ONO (tunneling oxide of 2 nm (*T_TOX_*)/nitride charge trapping layer of 4 nm (*T_N_*)/ blocking oxide of 6 nm (*T_BOX_*)) layers is responsible for excitation-inhibition function. The independently accessed inner core gate with a single oxide layer (Core gate oxide (*T_CGOX_*) of 20 nm) was utilized for the firing threshold voltage adjustment. The CSDG NW device has advantages of good reliability and process compatibility with complementary metal-oxide-semiconductor (CMOS) [15].

Device simulation was performed with the aid of Silvaco ATLAS (Santa Clara, CA, USA) simulation. A macro model (DYNASONOS) along with Fowler-Nordheim (F-N) tunneling, hot carrier injection, and Poole-Frenkel emission models were adopted to reflect behaviors of trapped charges in the nitride layer. To describe the spiking characteristics of the neuron, Fermi-Dirac statistics model, concentration-dependent, Shockley-Read-Hall generation and recombination model, Auger recombination model, bandgap narrowing model, field-dependent mobility model, and Lombardi’s mobility model were included. Impact ionization model which governs the firing characteristic of the neuron device and band-to-band-tunneling model which dominates the leaky property of the single transistor neuron were also included. Energy band diagrams were extracted along the channel at 1 nm depth from the interface between silicon and tunneling oxide.

## 3. Results and Discussion

**Figure 2:**
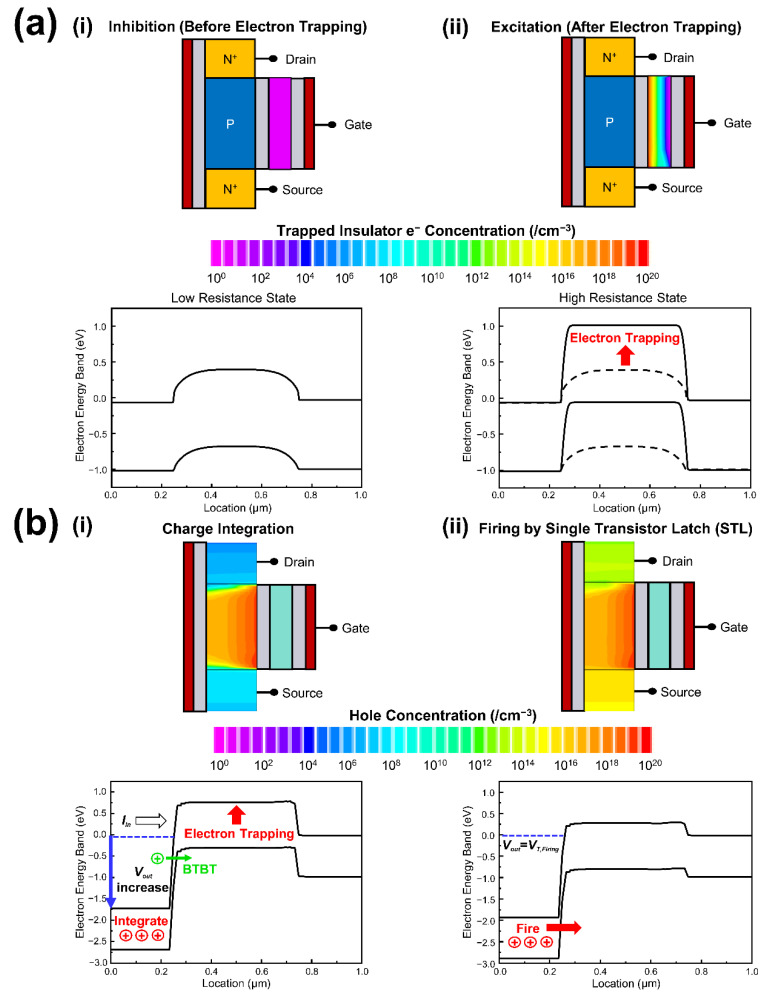
(**a**) Energy band diagrams and contour plots of trapped charges in nitride layer under inhibition mode and excitation mode; (**b**) Energy band diagrams and contour plots of hole concentration under charge integration process and firing process.

Figure 2a shows the energy band diagrams and the contour images of trapped electron concentration in nitride layer before and after electron trapping. Trapped electrons in nitride layer determine excitatory–inhibitory function of neuron. Before electrons were trapped in nitride layer, the device was in the inhibition mode since the neuron device was at a LRS as shown in Figure 2a(i), due to a low energy barrier at the channel between the source and drain. Because of the low resistance, current directly flowed through the channel when input current was applied to the drain. As a consequence, charges cannot be integrated in drain and a LIF operation was inhibited. By trapping electrons in nitride layer, the inhibition mode was switched to the excitation mode. When some electrons were trapped in the nitride layer by a shell gate voltage of 18 V for 10ms, built-in potential barrier was raised from the original level (dashed line) to a high level (straight line) so the resistance state was shifted from the LRS to the HRS (Figure 2a(ii). In this case, charges can be integrated by the input currents in the drain and the proposed device can serve ‘leaky integration’ before firing. Due to the non-volatility of the trapped charges, the energy consumption of the CSDG NW neuron was smaller than previous single transistor neuron devices which required continuous gate biasing [12].

Figure 2b shows the transient energy band diagrams and contour images of hole concentration during the LIF operation. Under the excitation mode, charges were accumulated in parasitic capacitance of drain with representing an increased output voltage when constant input current of 10 pA was applied to the drain as shown in Figure 2b(i). During the charge integration, the band-to-band-tunneling which is the dominant mechanism that determines the leaky characteristic of the single transistor neuron device was observed with the rate of 4.985 × 10^18^ at the junction between body and drain [24] (Figure 2b(i)). Although there was the leaky path, the output voltage was continuously increased by the input. By the STL phenomenon, the accumulated charges were suddenly fired when the output voltage reached to the firing threshold voltage (*V_T,Firing_*). Under the *V_T,Firing_*_,_ many holes were generated by impact ionization in the channel region and the channel holes lowered the potential barrier (Figure 2b(ii)). Due to a low energy barrier, more carriers were injected from source to body and promoted impact ionization again. By this positive feedback, the resistance state was abruptly shifted from the HRS to the LRS, and the charge firing was induced.

**Figure 3:**
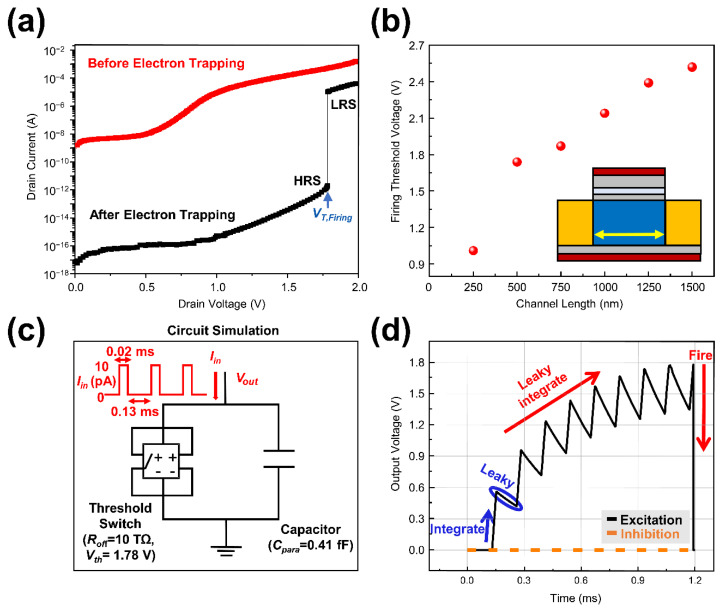
(**a**) *I_D_−V_D_* curves of the CSDG NW neuron device under inhibition mode and excitation mode; (**b**) The firing threshold voltage depending on the channel length of the device; (**c**) Equivalent circuit of the CSDG NW neuron composed of a threshold switch and a parasitic capacitance; (**d**) Current pulse measurement result to confirm LIF operation of the CSDG NW neuron device.

Figure 3a shows the *I_D_−V_D_* curves of the neuron device. Before electron trapping, the drain current flowed regardless of the drain voltage since the neuron was at LRS. After electron trapping, current did not flow at a low drain voltage range since the neuron was at HRS. When the drain voltage reached to the firing threshold voltage of the 1.74 V, the HRS neuron device was switched to the LRS and drain current abruptly flowed by the STL phenomenon. To confirm low power feasibility, the power consumption of CSDG NW neuron device was extracted. The peak power consumption when the firing process occurs was calculated by multiplying peak source current and peak output voltage *V_out_.* It was founded that CSDG NW neuron consumed a peak power of 0.18 μW. Figure 3b shows the effect of the channel length on the firing threshold voltage. The firing threshold voltage was affected by channel length since the lateral electric field can be approximated to *(V_D_−V_S_)/L_ch_*. Increased lateral electric field can induce a higher impact ionization and result in a decreased latch voltage. Therefore, the firing threshold voltage of the neuron device was decreased from 2.52 V to 1.01 V as the channel length decreased from 1500 nm to 250 nm. Note that the firing threshold voltage changed a lot at channel length of 250 nm due to the short channel effects.

To confirm output voltage spike generated by LIF operation, the CSDG NW neuron was modeled as a voltage-controlled threshold switch and a parasitic capacitor using a LTspice simulation (Analog Devices, Wilmington, NC, USA), as shown in Figure 3c. On the basis of measured data from the device simulator, the parasitic capacitance value and the firing threshold voltage value were fixed to 0.41 fF and 1.78 V, respectively. At the same node, voltage sensing and switching condition checking were conducted and *R_off_* (Resistance value of the HRS) was set to 10 TΩ. The input square pulse was applied to the neuron device with a peak of 10 pA and period of 0.15 ms. Figure 3d shows the leaky integrate-and-fire operation of the CSDG NW neuron device. During the first input pulse, charges were accumulated in parasitic capacitance while representing an increased output voltage. After the charge integration and before the next input pulse was applied to the device, the output voltage was decayed owing to its leaky characteristics. Under the repetitive input pulses, the output voltage was increased by the iterative leaky integrate process until the firing. When output voltage reached to the firing threshold voltage of 1.74 V, the accumulated charges in parasitic capacitance was discharged abruptly and firing was occurred. The averaged power consumption within one spike can be extracted by dividing the energy in one spike by the spiking period. The CSDG NW neuron device consumed average power of 8.8 pW for generating a single spike signal at a constant input current (*I_in_*) of 10 pA.

**Figure 4:**
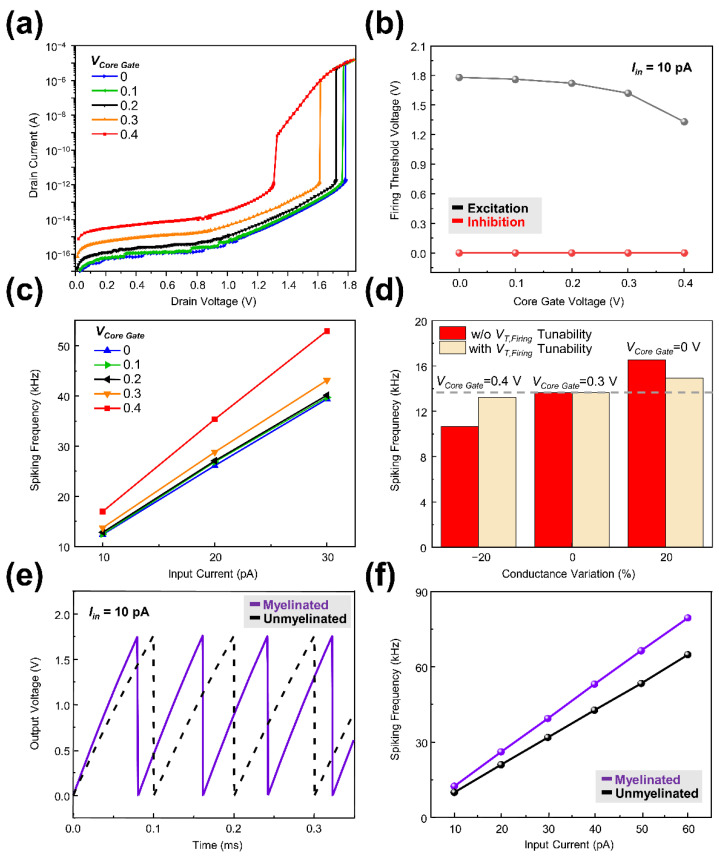
(**a**) *I_D_−V_D_* curves of the CSDG NW neuron device under different core gate bias; (**b**) firing threshold voltage of the CSDG NW neuron device under different core gate bias; (**c**) Spiking frequency of the CSDG NW neuron device depending on the core gate bias and the input current; (**d**) Spiking frequency with and without tunable firing threshold voltage under conductance variations of −20%, 0%, and 20%; (**e**) Comparison of the output voltage between myelinated neuron and unmyelinated neuron; (**f**) Spiking frequency of the myelinated and unmyelinated neuron depending on the input current.

The independently accessed core gate can be applied to tune the firing threshold voltage. The extra bias can be applied to the core gate for tuning the firing threshold voltage and this value can be modulated by controlling the thickness of the core gate oxide. Figure 4a shows the *I_D_−V_D_* curves of the neuron device under different core gate bias. The shell gate voltage of 18 V for 10ms was applied to the device and core gate bias was increased from 0 V to 0.4 V with the bias step of 0.1 V. By the increment of the core gate bias, more carriers were injected into the channel and promoted impact ionization. In result, the firing threshold voltage was lowered from 1.78 V to 1.33 V as shown in Figure 4b [24]. Under the inhibition mode, the spiking output voltage was not observed regardless of the core gate bias. In Figure 4c, the spiking frequency of the neuron was investigated under various core gate bias and input current. In earlier research, the spiking frequency of the LIF neuron was modeled by the following Equation (1) [21]:

(1) $$f=\frac{1}{\int_{0}^{V_{T,firing}}\left(\frac{C_{par}}{I_{in}-\frac{V_{out}}{R_{off}}}\right)dV_{out}}$$

Note that the spiking frequency is the function of the firing threshold voltage and the input current. Under the same constant input current of 10 pA, the spiking frequency of neuron was increased from 12.372 kHz to 16.955 kHz as core gate bias increased from 0 V to 0.4 V due to a lowered firing threshold voltage as shown in Figure 4c. Furthermore, with a fixed core gate bias, the spiking frequency of neuron was increased as the input current amplitude increased from 10 pA to 30 pA. This tunable firing threshold voltage can be applied to various applications. First, a random conductance variation of synapse before learning can be compensated. Due to the process-induced variations, synaptic devices can suffer from a random conductance variation before learning. Even 20% variation of the synapse conductance (Input current) can change spiking frequency of a neuron which is connected to the synapses by 3 kHz from the original state (0% variation of synapse conductance) (Figure 4d). If neuron devices have a firing threshold voltage tunability, each neuron can be controlled to generate same spiking frequency under the same input for the stable operation (Figure 4d). This firing threshold voltage tunability can be applied to not only “before learning” process but also “during learning” process. In many cases, homeostasis function which regulates firing rate of abnormal neuron is required in the learning process for the stable and effective learning. The implementation of homeostasis function in learning process can be facilitated by using the aforementioned fine tuning of firing threshold voltage in CSDG NW neuron device. For the actual implementation of homeostasis function, an additional circuit is needed to read abnormal spiking frequency and transmit appropriate firing threshold voltage adjustment signal.

In a biological neuron, membrane capacitance can be changed using myelin sheath. A myelinated neuron with smaller membrane capacitance allows the faster transmission of information compared with an unmyelinated neuron with larger capacitance. In vertical CSDG NW neuron device, the parasitic capacitance of drain was smaller than source due to the inherent asymmetry of S/D which is originated from the vertical structure, i.e., different size and doping concentration of top of the pillar (drain) and bottom of the pillar (source). Therefore, higher spiking frequency was obtained when the input current was applied to the drain region compared with the case where input current was applied to the source region because parasitic capacitance of source (0.51 fF) is larger than that of drain (0.41 fF) (Figure 4e,f). This frequency variability is very similar to the myelination of a biological neuron. The former case can be considered as myelinated state and the latter case can be thought as unmyelinated state. Under the same input current of 10 pA, the spiking frequencies of myelinated neuron and unmyelinated neurons were 12.39 kHz and 9.9 kHz, respectively. At the input current of 60 pA, the spiking frequencies of myelinated and unmyelinated neurons showed a huge difference of 14.63 kHz. Thus, the CSDG NW neuron device can be myelinated for the case that requires high speed computing rather than low power consumption or can be unmyelinated for the case that requires low power consumption rather than high speed computing by simply switching the input nodes (source to drain or drain to source for myelinated neuron and unmyelinated neuron, respectively) of neuron device. 

**Figure 5:**
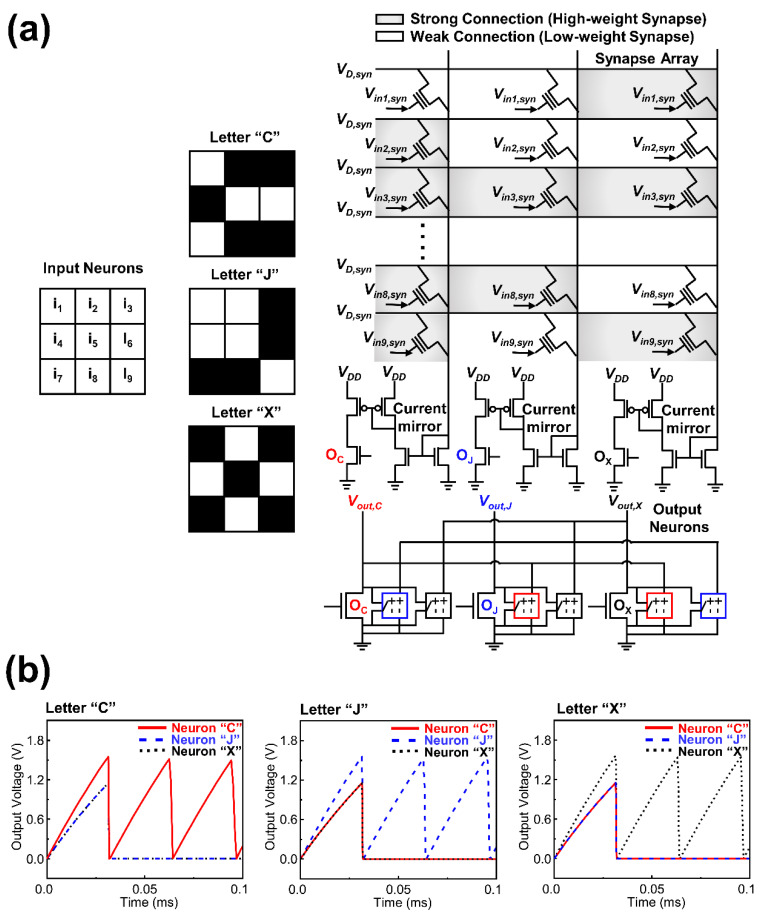
(**a**) Input images for letter recognition with 3 × 3 black-and white pixels and circuit configurations for the neural network system; (**b**) Results of the letter recognition.

The neuromorphic hardware is widely used to recognize various images such as patterns, objects, and letters. To demonstrate the application of the CSDG NW neuron, letter recognition system was established using a circuit simulation. Figure 5a shows the neuromorphic system to distinguish three input letters (“C”, “J”, and “X”) comprising 3 × 3 black-and-white pixels. The neuromorphic system was composed of 9 input neurons, 9 by 3 input synapses, and 3 output neurons. The input neurons were labeled from “i_1_” to “i_9_” corresponding to each pixel (Figure 5a). The input neurons generated input voltages (*V_in,syn_*) of 0 V for the white pixel and 1 V for the black pixel. The synapse array (9 by 3) with a strong connection (Gray color) and a weak connection (White color) was established between input and output neurons assuming precise leaning for the letter recognition had been completed. The CSDG NW synapse device was modeled with a three-terminal MOSFET (Channel length (*L_ch_*) = 500 nm and channel width (*W_ch_*) = 200 nm). The drain voltage of 1 V was applied to the device and threshold voltages of highly weighted, and lowly weighted synapse were set to 0 V, and 1V, respectively. Resultantly, the conductance values of highly weighted, and lowly weight synapse were 2 nS and 0.2 pS, respectively. The output currents from the synapse were summed and 400 fold scaled down by the current mirror and transferred to the neuron. Three output neurons (“O_C_”, “O_J_”, and “O_X_”) with the firing threshold voltage of 1.62 V were used to recognize each corresponding letter. The output neurons were connected to each other to realize the lateral inhibition as depicted in Figure 5a. The equivalent circuit model for the neuron device in Figure 3c was used for these output neurons. By using the aforementioned neuromorphic system including pre-trained synaptic weight and the proposed neuron device, letter recognition capability was verified. When input letter “C” was applied to the neuromorphic system, output neuron “O_C_” was excited and output neurons of “O_J_” and “O_X_” were inhibited. The neuromorphic system also successfully recognized the input letter in the cases of input letters “J” and “X” as shown in Figure 5b.

**Figure 6:**
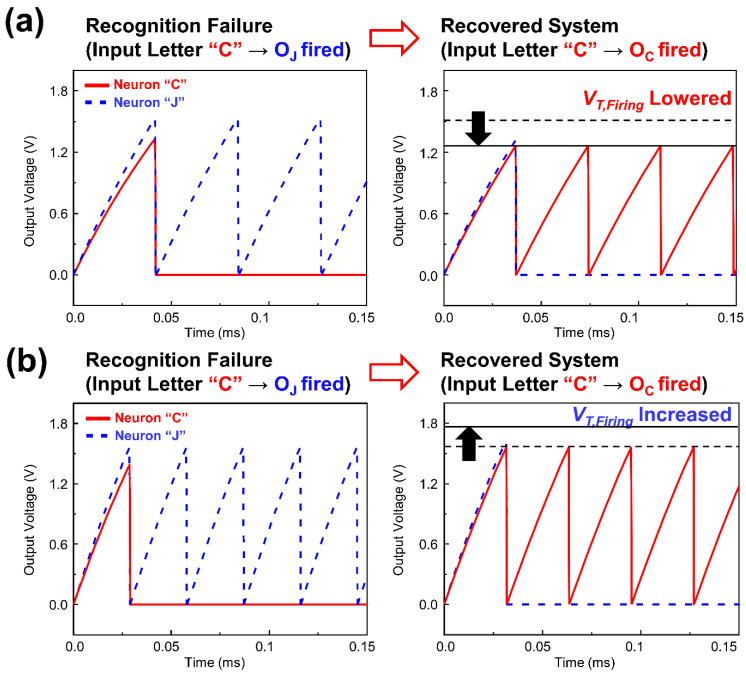
Letter recognition results of neuromorphic system before and after tuning firing threshold voltage when the threshold voltage of (**a**) high weight synapses connected to “O_C_” abnormally increased or (**b**) low weight synapses connected to “O_J_” abnormally decreased under the input letter “C”.

Although it was confirmed that SNN with the proposed neuron could successfully infer the letter patterns initially, the fact that synaptic devices can suffer from the conductance variations caused by endurance problem should be carefully considered. By unwanted conductance variations after the learning, inference error can be caused. This problem can also be fixed by the aforementioned firing threshold voltage tunability of the proposed neuron device. Assuming that threshold voltage of all the highly weighted synapses which connected to the “O_C_” was abnormally increased from 0 V to 0.2 V, the input current from the synapses to the “O_C_” was decreased because of the increased threshold voltage of the synapses and other output neuron “O_J_” fired before “O_C_” (Figure 6a). Therefore, the “O_C_” was inhibited by the output signal from “O_J_” and neuromorphic system failed to correctly recognize the letter “C” in the inference process. By lowering the firing threshold voltage of the output neuron “O_C_” from 1.62 V to 1.33 V, this inference error was simply corrected while forcing the output neuron “O_C_” to be fired in advance to the output neuron “O_J_”, and to inhibit other output neurons (Figure 6a). In the similar manner, the proposed neuron device could deal with the error caused by the synaptic weight variation in opposite polarity. When the threshold voltage of all the lowly weighted synapses connected to the “O_J_” was abnormally decreased from 1 V to −0.1 V, the input current from the synapses to the “O_J_” was also increased abnormally (Figure 6b). With the input letter “C”, the output neuron “O_J_” fired before “O_C_” reached to the firing threshold voltage and neuromorphic system failed to recognize the letter “C” in inference process. By increasing firing threshold voltage of “O_J_” from 1.62 V to 1.78 V, the output neuron “O_C_” fired before “O_J_” reached to the firing threshold voltage and successfully inhibited the output neuron “O_J_” (Figure 6b). As a consequence, stable letter recognition was realized by fine tuning of firing threshold voltage. To implement actual firing threshold voltage tuning, an additional circuit is required to receive the output voltage of the neuron for reading abnormal spiking frequency and transmit extra bias to the core gate.

## 4. Conclusions

A vertical core–shell dual-gate nanowire based single transistor neuron was demonstrated and verified by TCAD simulation. This vertical-typed device has an advantage for high density SNN hardware. The excitation-inhibition function was realized with a reduced energy consumption using a charge trapping shell gate with a nitride charge trapping layer. An independently accessed core gate was used to adjust the firing threshold voltage for a reliable neuromorphic system. Due to the asymmetric nature of source and drain in vertical nanowire structure, the myelination function which controls transmission rate was also realized. A system-level validation was conducted by the letter recognition test using a circuit simulation which reflects the device characteristics of neuron.
